# Supplementary material for: Systematic Review of Mind–Body Modalities to Manage the Mental Health of Healthcare Workers during the COVID-19 Era
Source: Healthcare (Basel). 2022 Jun 1;10(6):1027. doi: 10.3390/healthcare10061027 (PMC9222815; doi:10.3390/healthcare10061027)
Supplement: Supplementary file 1 [file healthcare-10-01027-s001.zip › healthcare-1741144-supplementary.pdf]

Supplement S1: PRIMSA 2020 checklist

Section and Topic	Item #	Checklist item	Location where item is reported
**TITLE**			
Title	1	Identify the report as a systematic review.	1
**ABSTRACT**			
Abstract	2	See the PRISMA 2020 for Abstracts checklist.	1
**INTRODUCTION**			
Rationale	3	Describe the rationale for the review in the context of existing knowledge.	1-2
Objectives	4	Provide an explicit statement of the objective(s) or question(s) the review addresses.	2
**METHODS**			
Eligibility criteria	5	Specify the inclusion and exclusion criteria for the review and how studies were grouped for the syntheses.	2-3
Information sources	6	Specify all databases, registers, websites, organisations, reference lists and other sources searched or consulted to identify studies. Specify the date when each source was last searched or consulted.	2
Search strategy	7	Present the full search strategies for all databases, registers and websites, including any filters and limits used.	2<br>Supple 2
Selection process	8	Specify the methods used to decide whether a study met the inclusion criteria of the review, including how many reviewers screened each record and each report retrieved, whether they worked independently, and if applicable, details of automation tools used in the process.	3
Data collection process	9	Specify the methods used to collect data from reports, including how many reviewers collected data from each report, whether they worked independently, any processes for obtaining or confirming data from study investigators, and if applicable, details of automation tools used in the process.	3
Data items	10a	List and define all outcomes for which data were sought. Specify whether all results that were compatible with each outcome domain in each study were sought (e.g. for all measures, time points, analyses), and if not, the methods used to decide which results to collect.	3
	10b	List and define all other variables for which data were sought (e.g. participant and intervention characteristics, funding sources). Describe any assumptions made about any missing or unclear information.	3
Study risk of bias assessment	11	Specify the methods used to assess risk of bias in the included studies, including details of the tool(s) used, how many reviewers assessed each study and whether they worked independently, and if applicable, details of automation tools used in the process.	3
Effect measures	12	Specify for each outcome the effect measure(s) (e.g. risk ratio, mean difference) used in the synthesis or presentation of results.	3-4
Synthesis methods	13a	Describe the processes used to decide which studies were eligible for each synthesis (e.g. tabulating the study intervention characteristics and comparing against the planned groups for each synthesis (item #5)).	3-4
	13b	Describe any methods required to prepare the data for presentation or synthesis, such as handling of missing summary statistics, or data conversions.	3-4
	13c	Describe any methods used to tabulate or visually display results of individual studies and syntheses.	3-4
	13d	Describe any methods used to synthesize results and provide a rationale for the choice(s). If meta-analysis was performed, describe the model(s), method(s) to identify the presence and extent of statistical heterogeneity, and software package(s) used.	3-4
	13e	Describe any methods used to explore possible causes of heterogeneity among study results (e.g. subgroup analysis, meta-regression).	3-4
	13f	Describe any sensitivity analyses conducted to assess robustness of the synthesized results.	3-4
Reporting bias assessment	14	Describe any methods used to assess risk of bias due to missing results in a synthesis (arising from reporting biases).	4
Certainty	15	Describe any methods used to assess certainty (or confidence) in the body of evidence for an outcome.	N/A

Section and Topic	Item #	Checklist item	Location where item is reported
assessment			
**RESULTS**			
Study selection	16a	Describe the results of the search and selection process, from the number of records identified in the search to the number of studies included in the review, ideally using a flow diagram.	4
	16b	Cite studies that might appear to meet the inclusion criteria, but which were excluded, and explain why they were excluded.	4
Study characteristics	17	Cite each included study and present its characteristics.	4-5
Risk of bias in studies	18	Present assessments of risk of bias for each included study.	7-9
Results of individual studies	19	For all outcomes, present, for each study: (a) summary statistics for each group (where appropriate) and (b) an effect estimate and its precision (e.g. confidence/credible interval), ideally using structured tables or plots.	10-13
Results of syntheses	20a	For each synthesis, briefly summarise the characteristics and risk of bias among contributing studies.	10
	20b	Present results of all statistical syntheses conducted. If meta-analysis was done, present for each the summary estimate and its precision (e.g. confidence/credible interval) and measures of statistical heterogeneity. If comparing groups, describe the direction of the effect.	10
	20c	Present results of all investigations of possible causes of heterogeneity among study results.	10
	20d	Present results of all sensitivity analyses conducted to assess the robustness of the synthesized results.	10
Reporting biases	21	Present assessments of risk of bias due to missing results (arising from reporting biases) for each synthesis assessed.	14
Certainty of evidence	22	Present assessments of certainty (or confidence) in the body of evidence for each outcome assessed.	N/A
**DISCUSSION**			
Discussion	23a	Provide a general interpretation of the results in the context of other evidence.	14
	23b	Discuss any limitations of the evidence included in the review.	15
	23c	Discuss any limitations of the review processes used.	15
	23d	Discuss implications of the results for practice, policy, and future research.	14-15
**OTHER INFORMATION**			
Registration and protocol	24a	Provide registration information for the review, including register name and registration number, or state that the review was not registered.	14
	24b	Indicate where the review protocol can be accessed, or state that a protocol was not prepared.	14
	24c	Describe and explain any amendments to information provided at registration or in the protocol.	14
Support	25	Describe sources of financial or non-financial support for the review, and the role of the funders or sponsors in the review.	16
Competing interests	26	Declare any competing interests of review authors.	16
Availability of data, code and other materials	27	Report which of the following are publicly available and where they can be found: template data collection forms; data extracted from included studies; data used for all analyses; analytic code; any other materials used in the review.	16

From: Page MJ, McKenzie JE, Bossuyt PM, Boutron I, Hoffmann TC, Mulrow CD, et al. The PRISMA 2020 statement: an updated guideline for reporting systematic reviews. BMJ 2021;372:n71. doi: 10.1136/bmj.n71

For more information, visit: http://www.prisma-statement.org/

Supplementary S2. Search strategies used in each database Medline via PubMed

	Searches	Results
#1	COVID-19[MH] OR SARS-CoV-2[MH] OR COVID-19[TIAB] OR SARS-CoV-2[TIAB] OR (wuhan[TIAB] AND coronavirus[TIAB]) OR 2019-nCoV[TIAB] OR 2019nCoV[TIAB]	194,212
#2	Nurses[MH] OR Nursing[MH] OR nurs*[TIAB]	676,511
#3	Physicians[MH] OR physician*[TIAB] OR doctor*[TIAB]	618,652
#4	"Health Personnel"[MH] OR "health personnel*"[TIAB] OR "healthcare worker*"[TIAB] OR "hospital staff*"[TIAB] OR "health manager*"[TIAB]	582,824
#5	"Mind-Body Therapies"[MH] OR Meditation[MH] OR Mindfulness[MH] OR Relaxation[MH] OR "Relaxation Therapy"[MH] OR "Autogenic Training"[MH] OR Yoga[MH] OR "Tai Ji"[MH] OR Qigong[MH] OR "Breathing Exercises"[MH] OR "Music Therapy"[MH] OR "Imagery, Psychotherapy"[MH] OR "Biofeedback, Psychology"[MH] OR mind-body[TIAB] OR meditation[TIAB] OR mindful*[TIAB] OR relaxation[TIAB] OR "autogenic training"[TIAB] OR yoga[TIAB] OR "Tai Ji"[TIAB] OR "Tai Chi"[TIAB] OR Taichi[TIAB] OR qigong[TIAB] OR "qi gong"[TIAB] OR breathing[TIAB] OR music[TIAB] OR "guided imagery"[TIAB] OR biofeedback[TIAB] OR prayer[TIAB] OR faith[TIAB]	316,813
#6	#1 AND (#2 OR #3 OR #4) AND #5	**286**

EMBASE via Elsevier

	Searches	Results
#1	'coronavirus disease 2019'/exp OR 'coronavirus disease 2019':ab,ti OR COVID-19:ab,ti OR 'Severe acute respiratory syndrome coronavirus 2'/exp OR 'Severe acute respiratory syndrome coronavirus 2':ab,ti OR SARS-CoV-2:ab,ti OR (wuhan:ab,ti AND coronavirus:ab,ti) OR 2019-nCoV:ab,ti OR 2019nCoV:ab,ti	206,599
#2	nurse/exp OR nursing/exp OR nurs*:ab,ti	808,550
#3	Physician/exp OR physician*:ab,ti OR doctors*:ab,ti	1,260,138
#4	'health care personnel'/exp OR 'health care personnel*':ab,ti OR 'health personnel*':ab,ti OR 'healthcare worker*':ab,ti OR 'hospital personnel'/exp OR 'hospital personnel*':ab,ti OR 'hospital staff*':ab,ti OR 'health manager*':ab,ti	1,768,563
#5	'mind-body therap*':ab,ti OR meditation/exp OR meditation:ab,ti OR mindfulness/exp OR mindfulness:ab,ti OR 'relaxation training'/exp OR relaxation:ab,ti OR 'autogenic training'/exp OR 'autogenic training':ab,ti OR yoga/exp OR yoga:ab,ti OR 'Tai Chi'/exp OR 'Tai Chi':ab,ti OR 'Tai Ji':ab,ti OR Taichi:ab,ti OR qigong/exp OR qigong:ab,ti OR 'qi gong':ab,ti OR 'breathing exercise'/exp OR 'breathing exercise':ab,ti OR 'music therapy'/exp OR 'music therapy':ab,ti OR 'guided imagery'/exp OR 'guided imagery':ab,ti OR biofeedback/exp OR biofeedback:ab,ti OR prayer:ab,ti OR faith:ab,ti	213,982
#6	#1 AND (#2 OR #3 OR #4) AND #5	**434**

CENTRAL

	Searches	Results
#1	MeSH descriptor: [COVID-19] explode all trees	771
#2	MeSH descriptor: [SARS-CoV-2] explode all trees	525
#3	(COVID-19 OR SARS-CoV-2 OR 2019nCoV OR (wuhan AND coronavirus)):ti,ab,kw	7,882
#4	#1 OR #2 OR #3	7,882

#5	MeSH descriptor: [Nurses] explode all trees	3,381
#6	MeSH descriptor: [Nursing] explode all trees	3,381
#7	(nurs*):ti,ab,kw	45,924
#8	#5 OR #6 OR #7	46,167
#9	MeSH descriptor: [Physicians] explode all trees	2,240
#10	(physician* OR doctor*):ti,ab,kw	60,258
#11	#9 OR #10	60,714
#12	MeSH descriptor: [Health Personnel] explode all trees	9,751
#13	(“health personnel*” OR “healthcare worker*” OR “hospital staff*” OR “health manager*”):ti,ab,kw	4,391
#14	#12 OR #13	11,990
#15	MeSH descriptor: [Mind-Body Therapies] explode all trees	6,747
#16	MeSH descriptor: [Meditation] explode all trees	671
#17	MeSH descriptor: [Mindfulness] explode all trees	1,095
#18	MeSH descriptor: [Relaxation] explode all trees	1,728
#19	MeSH descriptor: [Relaxation Therapy] explode all trees	2,013
#20	MeSH descriptor: [Autogenic Training] explode all trees	140
#21	MeSH descriptor: [Yoga] explode all trees	748
#22	MeSH descriptor: [Tai Ji] explode all trees	387
#23	MeSH descriptor: [Qigong] explode all trees	87
#24	MeSH descriptor: [Breathing Exercises] explode all trees	953
#25	MeSH descriptor: [Music Therapy] explode all trees	902
#26	MeSH descriptor: [Imagery, Psychotherapy] explode all trees	471
#27	MeSH descriptor: [Biofeedback, Psychology] explode all trees	1,462
#28	(mind-body OR meditation OR mindful* OR relaxation OR “autogenic training” OR yoga OR “Tai Ji” OR “Tai Chi” OR Taichi OR qigong OR “qi gong” OR breathing OR music OR “guided imagery” OR biofeedback OR prayer OR faith):ti,ab,kw	51,970
#29	#15 OR #16 OR #17 OR #18 OR #19 OR #20 OR #21 OR #22 OR #23 OR #24 OR #25 OR #26 OR #27 OR #28	55,036
#30	#4 AND (#8 OR #11 OR #14) AND #29 in Trials	**82**

CINAHL via EBSCO

	Searches	Results
#1	COVID-19[MH] OR SARS-CoV-2[MH] OR COVID-19[TX] OR SARS-CoV-2[TX] OR (wuhan[TX] AND coronavirus[TX]) OR 2019-nCoV[TX] OR 2019nCoV[TX]	79,851
#2	Nurses[MH] OR Nursing[MH] OR nurs*[TX]	2,005,867
#3	Physicians[MH] OR physician*[TX] OR doctor*[TX]	793,904
#4	“Health Personnel”[MH] OR “health personnel*”[TX] OR “healthcare worker*”[TX] OR “hospital staff*”[TX] OR “health manager*”[TX]	139,877
#5	“Mind-Body Therapies”[MH] OR Meditation[MH] OR Mindfulness[MH] OR Relaxation[MH] OR “Relaxation Therapy”[MH] OR “Autogenic Training”[MH] OR Yoga[MH] OR “Tai Ji”[MH] OR Qigong[MH] OR “Breathing Exercises”[MH] OR “Music Therapy”[MH] OR “Imagery, Psychotherapy”[MH] OR “Biofeedback, Psychology”[MH] OR mind-body[TX] OR	219,489

	meditation[TX] OR mindful*[TX] OR relaxation[TX] OR “autogenic training”[TX] OR yoga[TX] OR “Tai Ji”[TX] OR “Tai Chi”[TX] OR Taichi[TX] OR qigong[TX] OR “qi gong”[TX] OR breathing[TX] OR music[TX] OR “guided imagery”[TX] OR biofeedback[TX] OR prayer[TX] OR faith[TX]	
#6	#1 AND (#2 OR #3 OR #4) AND #5	**2,010**

AMED via EBSCO

	Searches	Results
#1	COVID-19[SU] OR SARS-CoV-2[SU] OR COVID-19[TX] OR SARS-CoV-2[TX] OR (wuhan[TX] AND coronavirus[TX]) OR 2019-nCoV[TX] OR 2019nCoV[TX]	199
#2	Nurses[SU] OR Nursing[SU] OR nurs*[TX]	19,742
#3	Physicians[SU] OR physician*[TX] OR doctor*[TX]	10,754
#4	“Health Personnel”[SU] OR “health personnel*”[TX] OR “healthcare worker*”[TX] OR “hospital staff*”[TX] OR “health manager*”[TX]	5,898
#5	“Mind-Body Therapies”[SU] OR Meditation[SU] OR Mindfulness[SU] OR Relaxation[SU] OR “Relaxation Therapy”[SU] OR “Autogenic Training”[SU] OR Yoga[SU] OR “Tai Ji”[SU] OR Qigong[SU] OR “Breathing Exercises”[SU] OR “Music Therapy”[SU] OR “Imagery, Psychotherapy”[SU] OR “Biofeedback, Psychology”[SU] OR mind-body[TX] OR meditation[TX] OR mindful*[TX] OR relaxation[TX] OR “autogenic training”[TX] OR yoga[TX] OR “Tai Ji”[TX] OR “Tai Chi”[TX] OR Taichi[TX] OR qigong[TX] OR “qi gong”[TX] OR breathing[TX] OR music[TX] OR “guided imagery”[TX] OR biofeedback[TX] OR prayer[TX] OR faith[TX]	10,494
#6	#1 AND (#2 OR #3 OR #4) AND #5	**3**

PsycARTICLES via ProQuest

	Searches	Results
#1	SU(COVID-19) OR SU(SARS-CoV-2) OR TIAB(COVID-19) OR TIAB(SARS-CoV-2) OR (TIAB(wuhan) AND TIAB(coronavirus)) OR TIAB(2019-nCoV) OR TIAB(2019nCoV)	723
#2	SU(Nurses) OR SU(Nursing) OR TIAB(nurs*)	1,799
#3	SU(Physicians) OR TIAB(physician*) OR TIAB(doctor*)	3,894
#4	SU(“Health Personnel”) OR TIAB(“health personnel*”) OR TIAB(“healthcare worker*”) OR TIAB(“hospital staff*”) OR TIAB(“health manager*”)	11,622
#5	SU(“Mind-Body Therapies”) OR SU(Meditation) OR SU(Mindfulness) OR SU(Relaxation) OR SU(“Relaxation Therapy”) OR SU(“Autogenic Training”) OR SU(Yoga) OR SU(“Tai Ji”) OR SU(Qigong) OR SU(“Breathing Exercises”) OR SU(“Music Therapy”) OR SU(“Imagery, Psychotherapy”) OR SU(“Biofeedback, Psychology”) OR TIAB(mind-body) OR TIAB(meditation) OR TIAB(mindful*) OR TIAB(relaxation) OR TIAB(“autogenic training”) OR TIAB(yoga) OR TIAB(“Tai Ji”) OR TIAB(“Tai Chi”) OR TIAB(Taichi) OR TIAB(qigong) OR TIAB(“qi gong”) OR TIAB(breathing) OR TIAB(music) OR TIAB(“guided imagery”) OR TIAB(biofeedback) OR TIAB(prayer) OR TIAB(faith)	4,208
#6	#1 AND (#2 OR #3 OR #4) AND #5	**1**

Supplementary S3. Methodological qualities of included studies Randomized controlled trials

Author	1. Random sequence generation	2. Allocation concealment	3. Blinding of participants and personnel	4. Blinding of outcome assessment	5. Incomplete outcome data	6. Selective reporting	7. Other sources of bias
**Fiol-DeRoque 2021**	Low	Unclear	Low	Unclear	Low	Low	Unclear
**Nourian 2021**	Low	Unclear	High	Unclear	High	Unclear	Low
**Sanadgol 2021**	Unclear	Unclear	High	Unclear	Low	Unclear	Low
**Thimmapuram 2021**	Low	Unclear	High	High	High	Unclear	Low
**Vajpeyee 2021**	Unclear	Unclear	High	Unclear	High	Unclear	Unclear

Non-randomized controlled trials

Author	**Q1** Randomized trial	**Q2** Method of randomization	**Q3** Allocation concealment	**Q4** Double blindng	**Q5** Assessor blinding	**Q6** Baseline similarity between the groups	**Q7** Overall dropout rate	**Q8** Differential dropout rate	**Q9** Adherence to the intervention	**Q10** Similar background treatments	**Q11** Valid and realiable outcomes	**Q12** Sample size	**Q13** Pre-specified analysis	**Q14** ITT analysis
**Emmanuel 2021**	No	NA	NR	No	NR	NR	Yes	Yes	NR	Yes	Yes	NR	CD	NA
**Franco 2021**	No	NA	NR	No	NR	No	Yes	Yes	NR	NR	Yes	NR	CD	No
**Luton 2021**	No	NA	NR	No	NR	NR	No	No	NR	NR	Yes	NR	Yes	No
**Ibrahim 2022**	No	NA	NR	No	NR	NR	Yes	Yes	NR	NR	Yes	Yes	CD	NA

Abbreviation. CD, cannot be determined; NA, not applicable; NR, not reported.

Note. Quality Assessment of Controlled Intervention Studies: 1. Was the study described as a randomized, randomized trial, randomized clinical trial, or RCT? 2. Was the method of randomization adequate (i.e., the use of randomly generated assignments)? 3. Was the treatment allocation concealed (so that assignments could not be predicted)? 4. Were the study participants and providers blinded to the treatment group assignments? 5. Were the participants assessing the outcomes blinded to the group assignments? 6. Were the groups similar at baseline in important characteristics that could affect outcomes (e.g., demographics, risk factors, and comorbid conditions)? 7. Was the overall dropout rate from the study at an endpoint of 20% or lower for the number allocated to treatment? 8. Was the differential dropout rate (between treatment groups) at the endpoint of 15 percentage points or lower? 9. Was there high adherence to the intervention protocols in each treatment group? 10. Were other interventions avoided or similar in the groups (e.g., similar background treatments)? 11. Were the outcomes assessed using valid and reliable measures and implemented consistently across all study participants? 12. Did the authors report that the sample size was sufficiently large to detect a difference in the main outcome between the groups with at least 80% power? 13. Were the reported outcomes or subgroups analyzed pre-specified (i.e., identified before analyses were conducted)? 14. Were all randomized participants analyzed in the group to which they were originally assigned, that is, did they use an intention-to-treat analysis?

Before-after studies

Author	**Q1** Description of the study question	**Q2** Eligibility criteria	**Q3** Representativeness of participants	**Q4** Adherence of pre-specified entry criteria	**Q5** Sample size	**Q6** Description of the intervention	**Q7** Pre-specified outcome	**Q8** Assessor blinding	**Q9** Loss to follow-up	**Q10** Statistical methods	**Q11** Interrupted time-series design	**Q12** Use of individual-level data
**Giordano 2020**	Yes	Yes	Yes	Yes	No	Yes	No	No	Yes	Yes	No	NA

**Klatt 2020**	Yes	No	Yes	CD	No	Yes	Yes	No	No	Yes	No	NA
**Coffey 2021**	Yes	No	No	CD	No	No	Yes	No	Yes	Yes	No	NA
**Divya 2021**	Yes	No	Yes	CD	No	Yes	Yes	No	Yes	Yes	Yes	NA
**Heeter 2021**	Yes	No	Yes	CD	No	Yes	Yes	No	No	Yes	No	NA
**Liu 2021**	Yes	Yes	No	Yes	Yes	Yes	Yes	No	Yes	Yes	No	NA
**Narayanan 2021**	Yes	Yes	Yes	Yes	No	Yes	No	No	Yes	No	No	NA
**Nijland 2021**	Yes	Yes	Yes	Yes	No	Yes	Yes	Yes	Yes	Yes	No	NA
**So 2021**	Yes	Yes	Yes	CD	No	No	Yes	No	Yes	Yes	No	NA

Abbreviation. CD, cannot be determined; NA, not applicable.

Note. Quality Assessment Tool for Before-After (Pre-Post) Studies With No Control Group: 1. Was the study question or objective stated clearly? 2. Were the eligibility criteria for the study population pre-specified and clearly described? 3. Were the study participants representative of those who would be eligible for the test/service/intervention in the general or clinical population of interest? 4. Were all eligible participants who met the pre-specified entry criteria enrolled? 5. Was the sample size sufficiently large to provide confidence in our findings? 6. Was the test, service, or intervention clearly described and delivered consistently across the study population? 7. Were the outcome measures pre-specified, clearly defined, valid, reliable, and consistently assessed across all study participants? 8. Were the people assessing the outcomes blinded to the participants' exposure or interventions? 9. Was the loss to follow-up after a baseline of 20% or less? Were those patients lost to follow-up in the analysis? 10. Did the statistical methods examine the changes in outcome measures from before to after the intervention? Were statistical tests performed that provided p-values for pre-to post-changes? 11. Were outcome measures of interest taken multiple times before and after the intervention (i.e., did they use an interrupted time-series design)? 12. If the intervention was conducted at a group level (e.g., a whole hospital, a community, etc.), did the statistical analysis take into account the use of individual-level data to determine effects at the group level?
